# Bread without Yeast

**Published:** 1859-03

**Authors:** 


### BREAD WITHOUT YEAST,

Salt, milk, salæratus, soda or any think else is made as follows, according to Elsie M. Emory, of Cardington, Ohio, as communicated through that delightful family paper the *Country Gentleman*, of Albany, New York:—Take boiling water, let it stand until the temperature is reduced below the scalding point, then stir in flour as thick as you can well beat it with a spoon. Set it in warm water kept at a proper temperature to promote fermentation, usually three or four hours. If it should become thin after standing a while, stir in a teaspoonful or two of flour, beating it occasionally until it commences to rise.—When light, put it with the flour, mixing up with water, kneading thoroughly; then make into loaves and put on tins to rise, keeping warm and bake as usual.

